# Blister Formation in Film Insert Moulding

**DOI:** 10.3390/mi11040424

**Published:** 2020-04-17

**Authors:** Timo Wöhner, Aminul Islam, Hans N. Hansen, Guido Tosello, Ben R. Whiteside

**Affiliations:** 1 Department of Mechanical Engineering, Technical University of Denmark, 2800 Kgs. Lyngby, Denmark; timo_woehner@hotmail.de (T.W.); hnha@mek.dtu.dk (H.N.H.); guto@mek.dtu.dk (G.T.); 2 The Centre for Polymer Micro and Nano Technology (Polymer MNT), University of Bradford, BD7 1DP Bradford, UK; b.r.whiteside@bradford.ac.uk

**Keywords:** film insert moulding, surface analysis, blister formation, simulation

## Abstract

The formation of blister in the injection moulded parts, especially in the film insert moulded parts, is one of most significant causes of part rejection due to cosmetic requirements or functionality issues. The mechanism and physics of blister formation for molded parts are not well-understood by the state-of-the-art literature. The current paper increases the fundamental understanding of the causes for blister formation. In the experiment, a membrane strip of 5 mm in width was overmoulded with Polypropylene (PP), which formed a disc-shaped part with a diameter of 17.25 mm and a thickness of 500 µm. To investigate the influence of the processing parameters, a full factorial design of experiments (DoE) setup was conducted, including mould temperature (T_m_), barrel temperature (T_b_), injection speed (V_i_) and packing pressure (P_p_) as variables. The degree of blistering at the surface was characterized by the areal surface roughness parameters Spk and Smr1, measured with a confocal laser microscope. The measurements were taken on the 10 mm long section of the membrane surface in the centre of the moulded part across the entire width of the film. In addition, the film insert moulding (FIM)-process was simulated and the average shrinkage of the substrate material under the membrane was investigated. Eventually, a method and processing window could be defined that could produce blister-free parts.

## 1. Introduction

In film insert moulding (FIM) a preformed film is inserted in the mould and subsequently overmoulded. This injection moulding technique is mainly used to create decorative plastic parts with a high-quality surface finish and enables one-step fabrication of plastic components with a decorated or functional surface. FIM parts can be found for example in automotive industry, medical technology, design and fashion products, household appliances, as well as for mobile phones and many other applications [1]. Apart from this, FIM has been used to add functional features like nano-patterned structures [2] or Radio Frequency Identification tags (RFID-tags) [3] to the surface of injection moulded parts. Furthermore, the use of FIM to overcome the mechanical problems of weld lines in glass fibre filled thermoplastics has been reported [4]. Despite its manifold applications, the number of scientific publications on the topic is quite low and many fundamental issues need to be addressed for the realization of defect free FIM and to exploit the full benefit of FIM.

One of the key areas of research on FIM is the effect of the change in the cooling rate of the part when a film is inserted. A plastic film insert decreases the heat conduction between melt and mould by acting as a barrier layer, which influences the filling and cooling behaviour of the part. The warpage resulting from these uneven cooling conditions was investigated in some publications such as [1] and [5,6,7]. Chen et al. [7] reported an asymmetric flow behaviour of Polypropylene when a film was inserted, where the mould side covered by the film showed a flow leading effect. Furthermore, they found that the film insert slows down the increase of the mould surface temperature under the film insert and reduces the maximum mould surface temperature reached under the film insert compared to a conventional moulding process with the same mould. The warpage caused by these asymmetric cooling conditions increases with an increase in film thickness and melt temperature and decreases with an increase in mould temperature. These results are in good agreement with those found by Kim, et al. [8] who investigated the increase in the asymmetry of the temperature distribution with increasing film thickness.

The warpage of FIM-parts can be reduced or even reversed by an annealing step when an unannealed film was used. According to Kim, et al. [6] this effect is caused by the relaxation of residual stresses from film production, which can lead to a negative coefficient of thermal expansion (CTE) of the inserted film. In semi-crystalline materials, the formation of an amorphous skin layer can be suppressed due to the reduced cooling rate in the sections covered by the film. Kim et al. [9] found a higher degree of crystallization on the side of the part covered by the film, while the uncovered side showed an amorphous skin layer. The same effect was found by Chen et al. [7] who described an increase in crystallinity and the size of the crystallites with a decrease in the cooling rate due to the film insert, which also leads to an increase in warpage. Warpage in FIM has been investigated in several pieces of research and is considered as one of the main bottlenecks associated with the FIM process [10,11,12,13].

Another area of interest in FIM research deals with the adhesion between the film insert and the injection moulded substrate. Leong et al. [14] stated that the bonding strength can be improved by increasing injection speed and packing pressure. An increase in the barrel temperature below a certain limit increases the bonding strength, while the effect of an increase in barrel temperature above this level was found to be negligible. In [15], they describe a reduced peel strength for very high injection speeds, which is explained by a reduced entanglement of polymer chains across the interface between film insert and substrate due to an increase in molecular orientation. An additional annealing step led to even lower peel strength due to an increase in crystallization and the resulting reduction in loose molecules which are available for entanglements across the interface.

**Figure 1:**
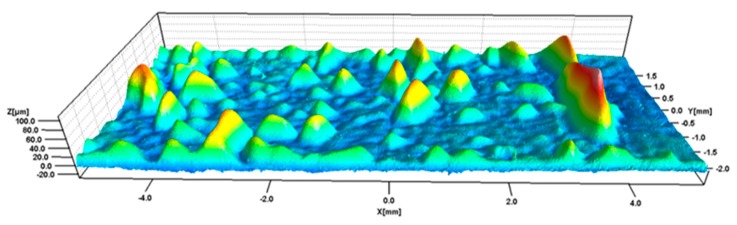
Blistered surface of the film insert after moulding (Z-axis scale 10:1). Moulding parameters: Mould temperature: 40 °C, barrel temperature: 200 °C.

In the current work the combination of a dual layer membrane made from a thin membrane layer, a non-woven support layer and polypropylene (PP) as an overmoulding material were tested for FIM. The ultimate goal of the work was to use the materials and process for the production of moulding-based methanol fuel cell (DMFC) containers, which are supposed to be used in hearing aid applications [16]. During this investigation, it was found that, for some combinations of the process, parameter blisters occurred in the membrane layer (see Figure 1). This phenomenon, to the author’s knowledge, has not been described in the literature before. For the investigation of the blisters, a confocal laser microscopy and film insert moulding simulations have been used. A full factorial design of experiments (DoE) was performed to define a process window that allows for blister free moulding and to investigate the relationship between the average shrinkage under the membrane found by means of simulation and the blistered surface analysis.

## 2. Materials and Methods 

### 2.1. Materials

In this work, a SABEU TRAKETCH^®^-membrane was used as film insert. This membrane has an overall thickness of 200 ± 20 μm. It consists of a 23 μm thick, porous PET-layer which has an oleophobic coating and a non-woven PP support. The pore size is 0.22 ± 0.02 μm and the pore-density is 270 × 10^6^ pores / cm². PP was used as substrate material (PP579S from SABIC, Riyadh, Saudi Arabia). This polypropylene grade shows a high melt flow index, provides high stiffness and low warpage tendency according to the supplier. Further background information on the material selection can be found in [17].

### 2.2. Part Design

**Figure 2:**
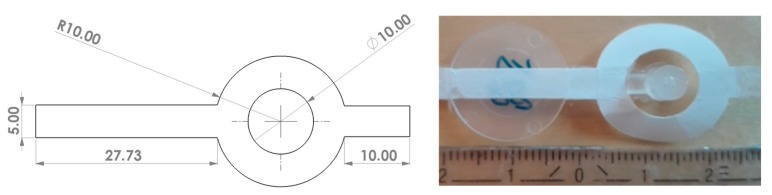
Design of the film insert blank (**left**, all dimensions in mm), film insert moulding (FIM)-part including sprue, runner, gate and untrimmed film insert (**right**).

The moulded part for this investigation was disc-shaped with a diameter of 17.25 mm and a thickness of 500 μm. In the centre of the disc, a 5 mm wide strip of the film insert was overmoulded which covered the whole diameter of the disc (see Figure 2). The film insert was aligned in the mould by placing it into a recess in the movable mould half, so that the support layer was overmoulded. It was fixed using double-sided adhesive tape at both ends of the film insert and additionally clamped between the mould halves. 

### 2.3. Moulding Experiments

**Figure 3:**
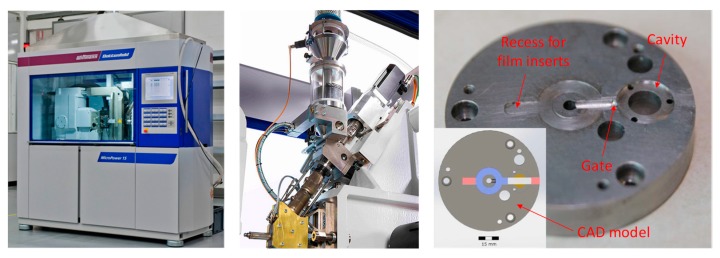
Wittmann–Battenfeld Micro Power 15 micro injection moulding machine used for the experiment (**left**), the injection unit of the of moulding machine (**middle**); and mould insert used in the tool to injection moulding of the FIM part (**right**).

For the moulding experiments, a Wittmann–Battenfeld Micro Power 15 micro injection moulding machine was employed (see Figure 3, which also shows the mould insert used for the FIM experiment). This machine uses a screw (diameter 14 mm) for plastification of the material and an injection plunger with a diameter of 5 mm for injecting the material. A full factorial DoE analysis was used to investigate the influence of the injection moulding parameters like mould temperature (T_m_), barrel temperature (T_b_), injection speed (V_i_) and packing pressure (P_p_). The chosen parameter levels can be found in Table 1.

The barrel temperatures were chosen to cover the temperature ranges given in the processing datasheet for the used polypropylene. The entire mould temperature range suggested in the datasheet could not be implemented, because the mould temperature control was based on electrical heating cartridges. This set-up did not allow for temperatures below room temperature. A temperature in the middle of the suggested T_m_ range was therefore used as low-level. A mould temperature of 60 °C is recommended in the datasheet for thick walled parts, but is used in these experiments to cover the range of recommended values as well as possible. To reduce the number of experimental runs, only two levels for the mould temperature were chosen. This decision was based on a paper by Chen et al. [7]. They found that the influence of mould temperature on warpage, crystallinity and crystal size shows a linear behaviour using PP in FIM. Therefore, a linear influence of T_m_ is expected in these experiments.

The packing pressure was set to 40%, 55% and 70% of the Maximum Injection Pressure (MIP). To define the MIP the DoE-runs showing the highest injection pressure were identified by means of simulations. The according processing parameters were used in the machine and the MIP was read from the machine as 180 bar. The packing time was set to four seconds throughout the set of experiments. This packing time is long enough to guarantee for a frozen gate. The maximum time for the gate freeze-off found in the simulations was below three seconds. Therefore, the packing time will have no influence on the experimental results. An additional cooling time of three seconds was used, to assure that the entire part is solidified. The influence of these two parameters can, therefore, be excluded. For each of the 54 runs of the DoE-table, five parts with membrane were moulded. Due to the manual insertion of the film insert, a long mould open time (30 s) had to be chosen. To stabilize the process conditions, five parts were moulded without membrane and rejected before a part with film insert was moulded.

### 2.4. Simulations

Computer modeling and simulation is revolutionizing manufacturing industries, allowing for optimizing product development process, improving resource management and enhancing the product quality [18,19]. Simulation aids the entire process, starting from component design, mold design, and manufacturing, material selection, molding conditions’ enhancement and ending with cooling optimization (shrinkage and warpage analysis). At present, it is possible to solve 3D problems by FEM including crystallization models to account for the changes in morphology that occur when semi-crystalline thermoplastics solidify [20,21]. For the current investigation, the “in-mould label overmoulding”-feature of Autodesk Mouldflow Simulation Insight 2016 was used to simulate the process sequence which contained filling, packing and cooling. This feature takes the influence of the film insert on flow and cooling behaviour into consideration [22]. The film insert was modelled as a 200 µm thick PP-film. The simulation model furthermore contained the mould, venting locations and the heating cartridges. A multi-scale mesh was used to achieve a sufficient mesh resolution at the edge of the film insert at acceptable computation times. It is assumed that the occurrence of the blisters is related to the shrinkage of the substrate material. If the shrinkage in the substrate is higher than the contraction of the film insert during the cooling phase of the process, a delamination between the film insert and the substrate material can occur [23]. For that reason, the average volumetric shrinkage of the substrate under the film insert was investigated in the simulations. Therefore, this shrinkage value was taken at 16 different locations under the film insert on the simulated part and the average of these values was used for the evaluation of the DoE.

### 2.5. Metrology

**Figure 4:**
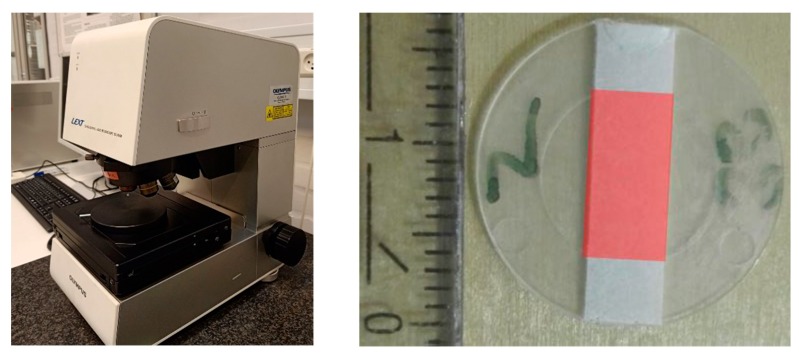
Olympus LEXT OLS 4000 3D measuring laser microscope used for the analysis (**left**), Measurement area of 5 × 10 mm² on the membrane in the center of the part—red area (**right**).

To characterize the degree of blistering of the membrane surface an area of 5 × 10 mm² in the centre of the disk-shaped part was measured using the 10× magnification lens of an OLYMPUS LEXT 4100 confocal laser scanning microscope (Olympus, Tokyo, Japan) in stitching mode in combination with post processing with SPIP™ image metrology software by Image Metrology A/S (version 6.4.3). This post processing routine contained steps for tilt compensation, warpage compensation and the application of a median filter to remove speckle noise. Eventually, an area of interest was defined (see Figure 4), the zero level of the measurement was set to the most frequent height level and the areal surface roughness parameters were calculated. To investigate the influence of the process parameters on the emergence of blisters the areal surface roughness parameters Spk (reduced peak height) and Smr1 (peak material portion) were evaluated. Spk, the reduced peak height, is a measure for the average height of the protruding peaks above the core roughness. The averaging process due to the calculation of this parameter reduces the influence of outliers. Smr1 is the material ratio above the core roughness and therefore a measure for the area covered by blisters. 

## 3. Results and Analysis 

In the following section, the results of the DoE are presented. The points in the graphs indicate the mean value of all observations obtained at the indicated level of the parameter. Error bars are used to indicate a 95% confidence interval based on the estimated standard error of the mean. For each experiment, 5 parts were molded, evaluated and average results are presented in the paper, hence the error bars indicate the standard deviation calculated from 5 data points.

### 3.1. Smr1

**Figure 5:**
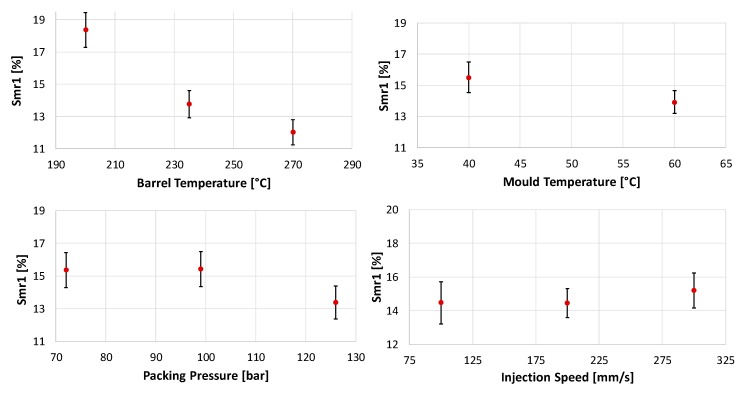
Main Effect of chosen parameters on the roughness parameter Smr1: effects of barrel temperature—T_b_ on Smr1 (**top left**); effects of mould temperature—T_m_ on Smr1 (**top right**); effects of packing pressure—P_p_ on Smr1 (**bottom left**); and effects of injection speed—V_i_ on Smr1 (**bottom right**).

**Figure 6:**
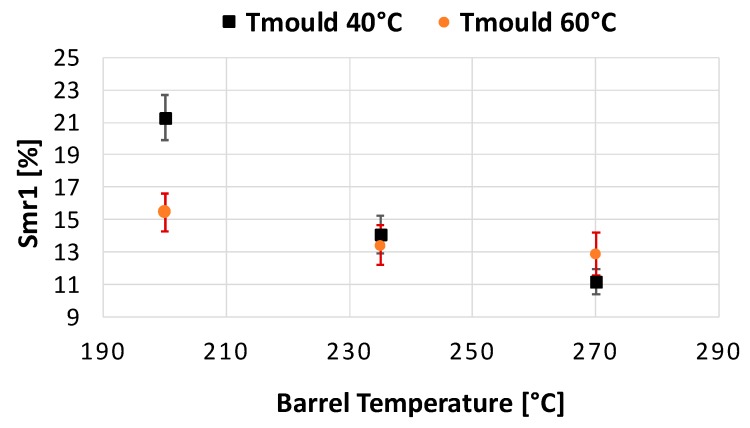
Two factor interaction of mould temperature (T_m_) and barrel temperature (T_b_) on Smr1.

According to the evaluation of the DoE, T_b_ had the greatest influence on the Smr1 value. Increasing T_b_ leads to a decrease in the portion of the surface, which is covered by blisters. In this evaluation, a 2^nd^ order regression model was used to identify the behaviour for the parameters with three levels. The regression model indicated a non-linear dependency between T_b_ and Smr1 presented in Figure 5. Furthermore, it was found that, T_m_ and P_p_ had a statistically significant influence on Smr1. For both, an increase in the input lead to a decreasing Smr1 value. Only V_i_ was found to have a non-significant effect at the chosen statistical significance level (α = 0.05). The only significant two-factor interaction found was between T_m_ and T_b_ (see Figure 6).

### 3.2. Spk

**Figure 7:**
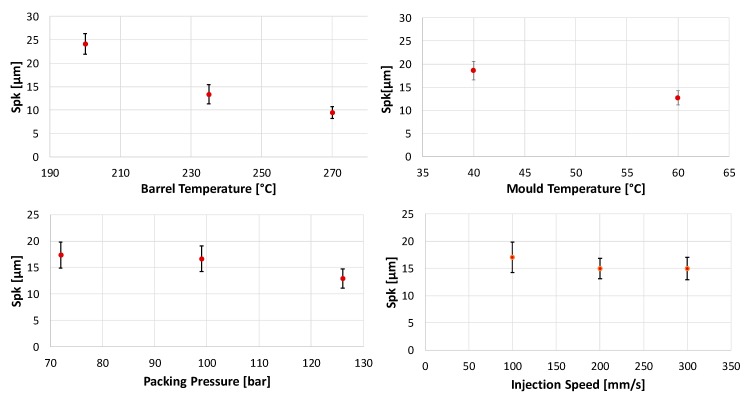
Main Effect of chosen parameters on the roughness parameter Spk: main effects of barrel temperature—T_b_ on Spk (**top left**); main effects of mould temperature—T_m_ on Spk (**top right**); main effects of packing pressure—P_p_ on Spk (**bottom left**); and main effects of injection speed—V_i_ on Spk (**bottom right**).

**Figure 8:**
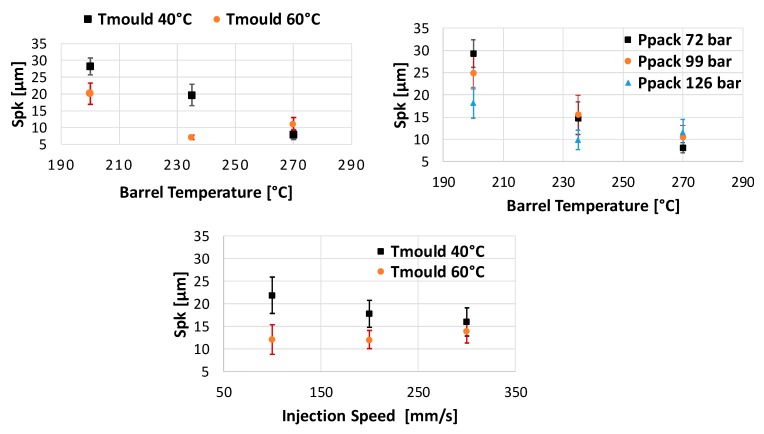
Two factor interactions for Spk: barrel temperature —T_b_ and mould temperature—T_m_ (**top left**); barrel temperature—T_b_ and packing pressure—P_p_(**top right**); injection speed—V_i_ and mould temperature—T_m_ (**bottom**).

The trends in the main effect plots are similar to those found for Smr1 (see Figure 7) This was expectable since higher blisters also lead to a larger share of the surface above the core roughness. According to the analysis of variance (ANOVA) for α=0.05, the only nonsignificant main effect is, as was found for Smr1, V_i_. In contrast to Smr1, Spk is influenced by three two-factor interactions. These are the interactions between T_b_ and T_m_, T_b_ and P_p_ and V_i_ and T_m_ (see Figure 8).

### 3.3. Average Volumetric Shrinkage under the Film Insert

**Figure 9:**
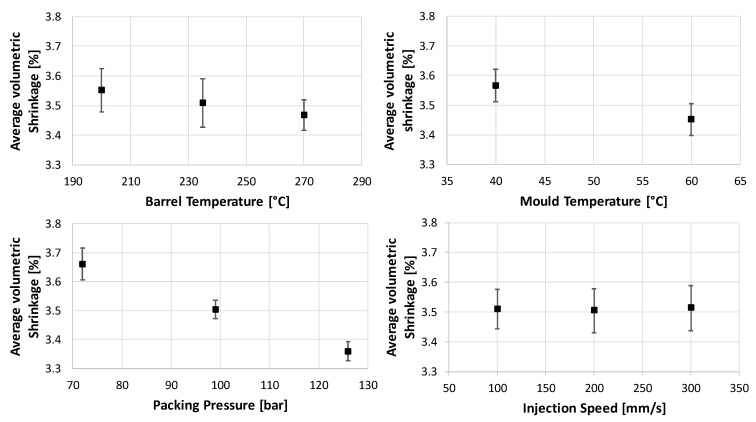
Main effects of chosen parameters on Average Volumetric Shrinkage (AVS): main effects of barrel temperature—T_b_ on AVS (**top left**); main effects of mould temperature—T_m_ on AVS (**top right**); main effects of packing pressure—P_p_ on AVS (**bottom left**); and main effects of injection speed—V_i_ on AVS (**bottom right**).

**Figure 10:**
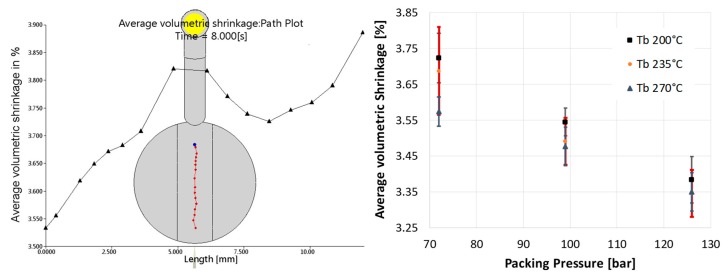
Average volumetric shrinkage path plot along the middle of the part (**left**), Two-factor interaction of T_b_ and P_p_ for “Average volumetric shrinkage” (**right**).

The evaluation of the simulation showed another order of significance of the process parameters (see Figure 9). The highest influence on this result was from P_p_, followed by T_m_. T_b_ and V_i_ did not show any significant influence. Unlike the results for the evaluation of Smr1 and Spk, all the parameters showed a linear influence in the simulation. A significant two-factor interaction was found between T_b_ and P_p_ (see Figure 10). Main effect and interactions plots obtained by simulation contain error bars in the following pictures. The evaluation is done based on an average value of a path in the center of the part, as shown in the left-hand side picture of Figure 10. The error bars show how much the values of the “Average Volumetric Shrinkage” are spread along this path. Since the data are based on the 54 runs of the “virtual” DoE, the values at the different points in the graphs show a certain distribution.

## 4. Discussion

According to the experimental results, the blister formation mainly depends on the process temperatures. A higher T_b_ mainly reduces the viscosity of the injected substrate material and increases the cooling time, while an increase in T_m_ increases the cooling time and reduces the thickness of the frozen layer at the interface between mould and melt and between melt and the film insert. This can lead to a more homogenous filling behaviour of the gaps between the fibres of the non-woven support layer, therefore, to a more homogenous cooling and shrinking behaviour under the membrane, which then would lead to a reduction in the blisters. 

In addition, higher temperatures lead to a longer effective packing phase, since the gate freezes off later. This extended packing phase could reduce the overall shrinkage of the part, and the emergence of blisters. Furthermore, this effect could be a possible explanation for the interaction between P_p_ and T_b_ found for Spk. This interaction could indicate that the packing result cannot be further improved by increasing P_p_ at the highest level of T_b_ because, at the extended effective packing time at the highest temperature level, even the lower levels of P_p_ are sufficient. 

The non-linearity found for T_b_ could be an effect of crystallization. At the prolonged cooling time at high temperatures, crystal growth can lead to an increased shrinkage, and to an increase in blister size and amount. This could also explain the interaction between T_m_ and T_b_ found for both Spk and Smr1. Another possible explanation for the non-linearity of T_b_ could be a stress-relaxation of the fibres of the non-woven support layer. When the melt is hot enough to raise the temperature of these fibers above the relaxation temperature, the relaxation of the frozen-in stresses of the fibres could cause their contraction. This could then lead to a delamination of the membrane from the support layer. 

**Figure 11:**
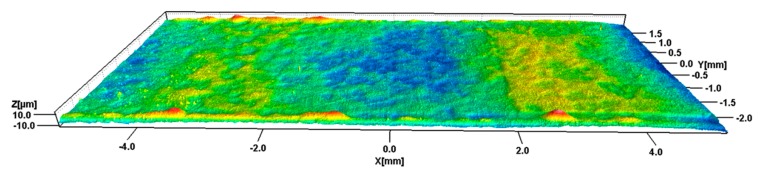
Part without blisters on the film insert surface (Z-axis scale 10:1) Molding parameters: T_m_: 60 °C, T_b_: 235 °C, P_p_: 126 bar, V_i_: 300 mm/s.

Eventually, a process window for blister-free parts could be defined. Parts moulded at the high level of T_m_ and P_p_ and the medium or the high level of T_b_ could be considered blister-free (see Figure 11) Comparing the experimental results with the simulation, a direct relation between the simulated parameter “Average Volumetric Shrinkage” and the experimental results could not be found. This is, however, no proof that the emergence of blisters is unrelated to the shrinkage of the injected substrate material under the film insert. Because of the different order of influence of the single parameters, the machine parameters recorded during the experiments were revised. It turned out that the switch-over point between the filling and packing phase was not hit properly at some of the runs. This late switch-over led to a significant increase in the maximum injection pressure and was found more often in runs with high levels of T_b_ and T_m_. The increase in the maximum injection pressure can lead to a kind of packing which then covers the effect of the actual packing phase. This can be a reason for the lower significance of the packing phase in the experimental results.

Further reasons for a poor reproduction of the experimental results by the simulation could be caused by the simplified model of the film insert, which did not consider the structure of the non-woven support layer, and therefore can lead to deviations from the experiments in the simulated filling behaviour and the simulated heat conduction. On top of that, there is no existing model for crystallinity for the used material. Even though a model for the calculation of crystallization effects based on [24] is implemented in the software, only few materials in the database provided the necessary input for the model when the study was conducted [25]. Therefore, the effects of crystallinity on the shrinkage are not considered in the simulations, but this is an important issue to achieve accurate simulation results [26,27,28]. Both these simplifications could lead to the linear behaviour found in the simulation, while T_b_ showed a non-linear influence in the experiments.

## 5. Conclusions and Outlook

In this paper, the blister formation process in FIM-parts was investigated. For that purpose, areal surface roughness parameters and simulations were used. It was found that the main influences on the formation of blisters were coming from the processing temperatures. An increase in T_m_ or T_b_ leads to a reduced blister height and a lower area portion covered by blisters. Eventually a process window for blister-free moulding could be defined for the material combinations used in the experiment. A direct relationship between the simulated shrinkage of the substrate material under the film insert could not be found, mainly due to problems with the switch-over point between the filling and packing phase. To improve the accuracy of the simulations, the experiments could be repeated with a material for which the switch-over point is easier to control. For the used material, the necessary back-pressure during the metering phase was in the range of the maximum back-pressure the machine could deliver. This is believed to be the reason for the problems with the switch-over point. Eliminating the switch-over problems would lead to a better comparability of simulations and experiments. Furthermore, an investigation of the crystallinity of the substrate material could provide information about the contribution of crystallization to the non-linear behavior found in the influence of T_b_. Future work should also focus on the combination of insert moulding and injection compression moulding to minimize the blister formation in FIM parts. Compression moulding has seen successfully used for the FIM process in several studies [29,30] and forms a strong basis for future research both for experimental and numerical activities.

## Figures and Tables

**Table 1:** Machine-settings for the moulding experiments.

Parameter	Low Level	Medium Level	High Level
T_m_	40 °C	-	60 °C
T_b_	200 °C	235 °C	270 °C
V_i_	100 mm/s	200 mm/s	300 mm/s
P_p_	72 bar	99 bar	126 bar
